# Consumers with bipolar disorder presenting to an Australian child and youth mental health service

**DOI:** 10.3389/fpsyt.2025.1514961

**Published:** 2025-03-27

**Authors:** Brett McDermott, Raja Sadhu, Mark Mayall

**Affiliations:** ^1^ Tasmanian Health Service (THS), Hobart, TAS, Australia; ^2^ University of Tasmania, Hobart, TAS, Australia; ^3^ Child Adolescent and Young Adult Services, Townsville University Hospital and Health Service, Townsville, QLD, Australia; ^4^ Child Adolescent and Young Adult Services, Townsville University Hospital and Health Service and James cook University, Townsville, QLD, Australia

**Keywords:** bipolar disorder, child, adolescent, mental health, service provision

## Abstract

**Introduction:**

The diagnoses reached for a consecutive set of consumers who presented to a public child and adolescent mental health service (CAMHS) in Australia were reviewed to assess the prevalence of bipolar disorder. Other presentations that had an element of mood elevation, specifically a diagnosis of a manic episode and cyclothymia, were also included.

**Methods:**

This study was a retrospective analysis of consecutive CAMHS consumers between 2014 and 2019.

**Results:**

Of 2131 consumers, the average age was 12.6 years and 2.4% of all first-episode diagnoses were either a manic episode, diagnosis of bipolar disorder, or cyclothymia. This represented approximately 20% of all mood disorder diagnoses. This group did not differ from other consumer presentations on gender, but they were approximately two years older at first episode. No consumer who presented when less than 12 years of age had a diagnosis of bipolar disorder or manic episode. The group were significantly lower on a measure of general psychosocial functioning. The cyclothymia/manic episode/bipolar disorder group was more likely to be admitted to a hospital mental health unit and at some time to be under the mental health act. Over the following five-year period, no patient with cyclothymia was diagnosed with a manic episode or bipolar disorder.

**Discussion:**

In summary, bipolar disorder is an uncommon diagnosis in children and adolescents attending CAMHS in an Australian regional city. However, these consumers are likely to have more psychosocial impairment and require more restrictive care.

## Introduction

Bipolar disorder (BD) is a chronic condition that has a lifetime prevalence of 1%–2.6%. The review of Cirone and colleagues reported that the prevalence of early-onset BD (i.e., onset under the age of 18 years) varied from 1.1% to 2.5% (1). The prevalence of sub-threshold symptoms of BD can be as high as 6.7% (2, 3).

BD in this age group has been linked to a more severe course of illness, worse prognosis, and higher suicidal rates (4). According to the Course and Outcome in Bipolar Disorder in Youth (COBY) study, the duration of manic episodes is longer in childhood-onset BD (78 weeks) compared with that in adolescence-onset BD. The onset of symptoms suggestive of BD varies in this age group. For example, summarizing three studies, those with an onset at 12 years or younger accounted for 5%–27% of patients with BD, while 23%–43% of patients had an onset between age 13 and 18 years (5–7).

A number of authors have noted that the BD presentations in the pediatric age group include long manic episodes and “affective storms” without the episodic course, which are normally found in adults (8). The existence of ultra-rapid and ultradian cycling consistent with BP-NOS (bipolar disorder not otherwise specified) has been described as common in very young children, indicating the complexity of the presentation of BD in this age group (9). Complicating this further are the mixed states/features that have also been reported to be common in children and adolescents presenting with BD (10).

BD in this age group is often comorbid with conditions such as attention deficit hyperactivity disorder (ADHD), oppositional defiant disorder (ODD)/conduct disorder (CD), depression, anxiety, and substance use disorder (11). ADHD, which is the most common comorbid condition, has been found to be present in 70%–95% of pre-adolescent youths and in 50% of adolescents. Similar rates for ODD and CD have been found, which were as high as 79% in youths with BD, and up to 77% of these patients have comorbid anxiety disorder. Different types of childhood trauma have been found to be frequently associated with BD and have been proposed to contribute to more severe symptomatology (12). Although there is no gender difference in the rates of early-onset BD, the onset of this condition occurs later in girls compared with boys, who also appear to have a higher frequency of comorbid ADHD (13).

Due to the significant mortality and morbidity associated with early-onset BD, along with its impact on life expectancy, diagnosing this condition early in life would be helpful for timely interventions. However, doubts have been raised about the real prevalence of this condition, as differences in the prevalence rates have been found between data from the USA and those from European countries, with an increased tendency of BD diagnosis in the USA. Reporting national datasets, Clacey et al. noted marked differences in the US rates of approximately 9–90 times those of comparison countries (14). It has been argued that for the pre-pubertal form of BD, which was proposed by the US researchers to explain chronic irritability and explosive temper, there was failure to consider the developmental history, the social context, and the clinical course while making these diagnoses (14). Emotional regulation, which is an important component of self-regulation, a part of human development, undergoes changes with age and tends to improve with time. However, different internal and external factors can change the trajectory of these changes and lead to difficulties in emotional regulation (15, 16), which might mimic the symptoms of early-onset BD.

The concept of BD in children comes with its own limitations; for example, the lack of appropriate self-appraisal system in children, the lack of appropriate reference points, the lack of appropriate language skills, and the limitations with regard to the source of information could all be barriers to making an appropriate diagnosis. On top of this, extrapolating the criteria for BD in adults in order to diagnose BD in children has failed to consider the developmental aspects of the presentations and the normal variations in the presentations in this age group (17).

As BD often starts with episodes of depression and an episode of mania does not manifest until subsequent presentations, the true nature of mental illness is not initially obvious, a phenomenon that makes the diagnosis of BD in children difficult. There is little current application of a biomarker to assist diagnosis. Neuroimaging studies have been conducted to differentiate unipolar depression from BD in children, which showed differences in the anterior cingulate cortex (ACC), the insula, and the dorsal striatum (18); however, the generalizability of these findings is limited. Furthermore, due to the recent reduction in importance of the mixed mood state in the Diagnostic and Statistical Manual of Mental Disorders, fifth edition (DSM-5), the diagnosis of BD in children and adolescents with mixed episodes, which has been found to be common in this age group, might be challenging. There have been proposals to not diagnose BD in children and to consider a diagnosis of BD only when the child reaches puberty (17). However, this presents a problem with regard to the management of those children who present with true BD early in their life. Delaying their diagnosis would deprive them of all the timely, evidence-based interventions. For a more detailed critique, see the reply of Connors (19).

The objective of this research was to add to studies that have applied rigorous diagnostic criteria to establish the prevalence of BD in children and adolescents by describing the prevalence of BD in an Australian Child and Adolescent Mental Health Service (CAMHS) based on routine clinical assessment practices. The specific goal was to report on the number of patients who present with a condition typified with an elevated mood, including the subset with a diagnosis of BD, to a public CAMHS. The secondary aims were to describe any demographic differences between the elevated mood groups and those with depression and to determine the number of individuals who convert from another presentation to BD over the 5-year period and any differences in this group in terms of service provision.

## Methods

### Procedure

The CAMHS cited in this research was the only public service provider for the mental health assessment and treatment of young people under the age of 18 in a regional city, Townsville, in the state of Queensland. This city is a regional center, with a population of 250,00, that has a referral catchment area inclusive of smaller towns and rural areas of approximately 700,000 people. CAMHS complied with the Australian minimum dataset for mental health services, which included the routine collection of demographic factors, diagnoses using the International Classification of Diseases—version 10 (ICD-10) classification system, and routine outcome measures. This study was a retrospective analysis of the CAMHS data collected from 2014 to 2019. The sample is a consecutive set of consumers less than 18 years of age who presented to any part of CAMHS—an outpatient clinic or the emergency department—or who were seen as a hospital patient by CAMHS consultation liaison practitioners. Initial assessment was offered to all consumers deemed to possibly have a mental health presentation of at least moderate severity, whose parents gave consent for assessment, or who were deemed sufficiently mature to provide their own consent. A lack of English competency or a low IQ were not part of the exclusion criteria. CAMHS is a public-funded tertiary service comprising three child and adolescent psychiatrists and a range of allied health and mental health nursing staff. There are 12 community clinical staff, supported by a day program and an inpatient unit. All patient assessments were presented at a multidisciplinary team meeting, and the ICD-10 diagnosis was made by a child and adolescent psychiatrist based on the information presented and/or their own personal assessment of the patient.

### Participants

The sample included 2,131 participants, of whom 50.1% were female patients and 49.9% male patients. Coding of gender was by parent report, and very few parents volunteered other gender descriptions. The average patient age at the beginning of an episode of care was 12.6 years (SD = 3.4 years). Approximately 18% of the sample identified as Aboriginal, Torres Strait Islander, or both. Overwhelmingly, participants were born in Australia (95.1%). The next highest recorded countries of birth were New Zealand (2.1%) and England (1.2%). The five most common ICD-10 diagnoses were childhood-onset conditions (41.1%), anxiety disorders (34.3%), mood disorders (13.8%), pervasive developmental disorder (7.6%), and behavioral syndromes (3.2%).

### Measures

The analysis included two measures that are mandated under the Australian Mental Health minimum dataset (20, 21). The Health of the Nation Outcome Scale Child and Adolescent version (HoNOSCA) is a clinician-reported measure including 15 items that cover a variety of domains, such as physical and mental health symptoms and school and disability items (22). It is scored on a scale from 0 (no problem) to 4 (severe to very severe problem), with a higher total score reflecting more severe problems. The review of Pirkis et al. suggests that the scale is suitable for research purposes (23). The Children’s Global Assessment Scale (CGAS) is also clinician-rated. It is based on descriptors that are inclusive of both symptoms and general functioning. High scores reflect an individual with higher functioning. A proxy measure for adverse childhood experiences (ACE-proxy) was calculated based on a conversion from the ICD-10 Z codes (factors influencing health status) recorded in the consumers’ electronic medical record. This methodology has been extensively discussed by Mayall et al. (24).

### Statistical analysis

The descriptive statistics are presented as percentages for categorical data and as mean and standard deviation for continuous data. The grouping variables included the ICD-10 Codes F30 (manic episode), F31 (BD), and F34 (cyclothymia). The latter is a mood disorder with persistent alternating periods of depression and elation, which is of less severity than BD. Another grouping variable was “any manic episode,” which is inclusive of individuals with only F30 and F31 diagnoses. The HoNOSCA used for the analyses was the 13 items summed to create a total score. Two categorical variables were analyzed with the chi-square test. For continuous dependent variables, a *t*-test was employed for nominal independent variables, while ANOVA was used for ordinal variables. Significance was set at *p* < 0.05. Data were analyzed with the statistical software package STATA (version 14, StataCorp, College Station, TX, USA).

## Results

### The first episode of care

During the first episode of care with CAMHS, mood disorders that feature some element of elevated mood (inclusive of a manic episode, a diagnosis of BD, or cyclothymia) were uncommon, representing only 2.4% of all first-episode diagnoses and 19.9% (49 of 246 patients) of patients with a mood disorder. Within this group (disorders with some feature of an elevated mood), the majority were a diagnosis of cyclothymia (39/246). Only six patients had a diagnosis of a manic episode and four of BD. When considering the group with any mood disorder diagnoses, there were no between-group differences in patient gender (*t* = 0.546, *p* = 0.759). Similarly, there was no difference in terms of indigenous status (*χ* ^2^ = 0.773, *p* = 0.670). Those in the bipolar/manic episode group were significantly older, by approximately 2 years (*F* = 14.99, *p* = 0.000) (see **Table 1**). From a categorical perspective, there were no individuals less than 12 years of age who presented with a bipolar or a manic episode. Two individuals with a cyclothymia diagnosis presented under 12 years. Of note is that the bipolar/manic episode patient group presented close to the upper age limit of the CAMHS. There was some suggestion that these groups also differed on important etiological factors in that the total number of ACE-proxy experiences differed, with the bipolar/manic group reporting significantly fewer ACEs than the other groups (*F* = 9.12, *p* = 0.000) (see **Table 1**).

**Table 1:** Comparison of bipolar/manic episode, cyclothymia, and depression groups.

Variable	Bipolar/manic Episode n (%)	Cyclothymia n (%)	Depression n (%)	Statistic	p
Gender male female	5/10 (50.0) 5/10 (50.0)	13/40 (32.5) 27/40 (67.5)	73/197 (37.0) 124/197 (63.0)	0.546^1^	0.759
Age (years) Mean (SD)	17.9 (2.13)	14.7 (2.0)	15.2 (1.54)	14.99^2^	0.000
Indigenous status Yes	1/9	8/39	32/166	0.773^1^	0.670
ACE total Mean (SD)	0.41 (0.59)	1.13 (1.01)	0.65 (0.87)	9.12^2^	0.000
Time 1 HoNOSCA Mean (SD)	16.2 (2.8)	17.5 (5.7)	15.9 (5.9)	1.16^2^	0.316
Time 1 CGAS Mean (SD)	45.0 (8.2)	51.5 (7.7)	55.5 (11.0)	3.87^2^	0.022

^1^X^2^ Test.

^2^ANOVA.

### Differences in the first episode of care outcome measures

There were no between-group significant differences in the HONOSCA scores when consumers presented at their first episode (*F* = 1.16, *p* = 0.316). There were significant differences in their overall functioning as measured by the CGAS. The general functioning scores of the bipolar/manic episode group were significantly lower compared with those of the other two groups (*F* = 3.87, *p* = 0.022) (see **Table 1**).

### Service provision and diagnostic categories

The number of service episodes across the 5-year data collection period was not observed to be a useful comparator given the significant difference in the age of the three groups at first presentation. Those in the bipolar/manic episode group were much more likely to quickly “age out” of the service. However, there were significant differences in the proportion of individuals in each diagnostic group who were admitted to a child and adolescent mental health inpatient unit. Overwhelmingly, those in the bipolar/manic episode group were the most admitted to hospital during an episode of care (85.7%) compared with those in the depression group (34.8%) and the cyclothymia group (15.4%). Consistent with the greater frequency of inpatient admissions, there was a statistically greater likelihood of admission being involuntary under the local Mental Health Act if the patient was in the bipolar/manic episode group (*F* = 41.32, *p* = 0.000) (see **Table 2**).

**Table 2:** Service provision-related variables across the data collection period.

Variable	Bipolar/manic Episode n (%)	Cyclothymia n (%)	Depression n (%)	Statistic X^2^	p
Inpatient admission	12/14 (85.7)	6/39 (15.4)	62/178 (34.8)	22.522	0.000
MHACT, Involuntary status	13/22 (59.1)	4/65 (6.1)	20/176 (11.4)	41.32	0.000

### Is cyclothymia a precursor of a later manic episode or a bipolar disorder diagnosis

There was no co-occurrence of a diagnosis of both cyclothymia and either BD or a manic episode in any of the patients. No patient with a cyclothymia diagnosis subsequently experienced a manic episode or had a diagnosis of BD. Three patients with an initial diagnosis of manic episode had a BD diagnosis during a subsequent episode of care.

## Discussion

There is a long tradition of empirical evidence that variations in diagnostic practices account for the differences in the prevalence of conditions (25). The methodology of this paper did not include a cross-country or cross-regional comparison of BD in children and adolescents. Rather, our aim was to describe the diagnoses for children and adolescents attending the CAMHS of a regional Australian city who had a diagnosis of BD or a manic episode based on the ICD-10 classification system (26). The findings demonstrate that psychiatrists within this service rarely make a diagnosis of BD. The rate of any diagnosis with a mania component in our sample (2.4% of all first-episode diagnoses) was of the same order of magnitude as the review findings of Cirone et al. (1). If a diagnosis is made, it is in consumers who are significantly older than other patients with a mood disorder. Indeed, our finding of no consumer presenting with bipolar/manic episode prior to the age of 12 years is not consistent with the results of the COBY study (13) but is more consistent with the critic of Duffy et al. (15). Furthermore, this patient group had higher levels of severity and acuity in that the group was significantly lower on a measure of global impairment. Moreover, the group is more likely to be admitted to the children and adolescent inpatient mental health unit rather than the outpatient services. When admitted, this group is more likely to be admitted under the Mental Health Act, strongly suggesting that clinicians deemed this group to be at more risk to themselves and/or others. These indices of severity (a requirement for inpatient care and use of the Mental Health Act) are consistent with the findings of Cicohn of a more severe illness course (4). There is some suggestion of other aetiological differences in mood disorders in children and adolescents given the significantly lower total number of adverse life experiences in the bipolar/manic group. A replication of this finding with a valid and reliable ACE measure would help clarify this result. There is a possibility of the underreporting of ACEs in these children and adolescents, which could have led to this finding. The other possibility is that genetic factors might have contributed more to the development of these conditions. We have not explored the extent of ACE exposures in the lives of the parents of these clients. There are possibilities that the epigenetic changes from ACE exposures in their parents that were passed on to these clients might have been contributory.

Given that the patients attending this CAMHS with a cyclothymia diagnosis were, on average, 2 years younger than those with a manic episode or BD, we investigated whether some of those with cyclothymia were subsequently reclassified with these more serious mental health presentations. In no case did this occur during the 2 years (based on the mean age of presentation) that these individuals could have represented to the service. Although our data did not suggest a relationship between these conditions, there are possibilities that conditions such as a chaotic family environment, posttraumatic stress disorder, emergence of puberty and the surge of sex hormones, and inappropriate peer influences might have overshadowed the symptoms of cyclothymia until more serious presentations such as mania manifested, leading to their presentations to the health service.

Clinicians make diagnoses that fit the diagnostic criteria of the classification system that they are trained to use or that their service mandates. Once made, these diagnoses facilitate the application of treatment research and are strongly linked to evidence-based intervention guidelines.

Considering the age of presentation and the presence of coexisting conditions such as a chaotic family environment, difficulties in parenting, difficulties in school adjustment, exposure to multiple types of psychological trauma, and presence of comorbid ADHD, among other neurodevelopmental disorders, the management of those conditions often became the priority and consumed most of the time-of-service delivery. Because of the possibility of other explanations behind their behavior, the diagnosis of BD might not have been considered, at least in some situations.

Practitioners in this service have applied evidence-based management of BD, for instance the use of mood stabilizer medication, to very few patients. Whether a broader definition of BD would lead to more patients receiving mood stabilizer medication and gaining benefit cannot be answered by this study but is worthy of inquiry. We can say that patients attending this service are protected from adverse medical events related to these medications.

### Limitations

This is a study of public patients in “real-world” settings. A more detailed clarification of the diagnoses using a structured diagnostic instrument is unlikely to be possible in such a busy public setting. Greater diagnostic rigor could be created by adding a mood (including elevated mood) symptom questionnaire. In the first instance, this could be with the older subset of patients.

## Conclusion

BD is a rare diagnosis in children and adolescents attending a public CAMHS in a regional Australian city. This is especially the case for individuals presenting when less than 12 years of age.

## Data Availability

The raw data supporting the conclusions of this article will be made available by the authors, without undue reservation.
